# Decoupling Effect of County Carbon Emissions and Economic Growth in China: Empirical Evidence from Jiangsu Province

**DOI:** 10.3390/ijerph19063275

**Published:** 2022-03-10

**Authors:** Yanli Ji, Jie Xue

**Affiliations:** 1 School of Mathematics and Statistics, Changshu Institute of Technology, Changshu 215500, China; 2 School of Economics, Hangzhou Dianzi University, Hangzhou 310018, China; xjsnow@hdu.edu.cn

**Keywords:** carbon emissions, economic growth, decoupling effect, Tapio elasticity model, environmental Kuznets curve

## Abstract

Under the pressure of low-carbon development at county level in China, this paper takes Jiangsu province as an example to analyze the relationship between economic growth and carbon emissions, aiming to provide a reference for the low-carbon development in Jiangsu and other regions in China. Based on the county-level panel data from 2000 to 2017, this paper uses the Tapio elasticity model and environmental Kuznets curve model, and focuses on the differences in regional economic development and the impacts of the 2008 global economic crisis. The results show that, in general, the decoupling effect of carbon emissions in Jiangsu counties has gradually increased during the study period. Since 2011, all counties achieved the speed decoupling, with more than half of them showing strong decoupling. The environmental Kuznets curves of carbon emissions in different income groups are established, and changed before and after the 2008 global economic crisis. In 2017, only 10 of the 53 counties were on the right side of the curve, realizing the quantity decoupling between the two. Therefore, to achieve a win–win situation between carbon emission reduction and economic growth, efforts should be made from the aspects of industrial structure and energy efficiency, and measures should be taken according to local conditions.

## 1. Introduction

Global warming, which is caused by greenhouse gas emissions (mainly carbon dioxide emissions), still remains one of the serious challenges facing human beings at present [1,2,3,4]. In order to ensure that the increase in global average temperature is kept within 2 °C from pre-industrial levels, countries around the world are actively implementing carbon emissions reduction actions [5,6,7]. China, as the world’s largest carbon emitter [8,9], is also making active and fruitful efforts. However, the situation in China’s counties is not optimistic. Due to different stages of economic development, China’s counties have undertaken many backward-capacity enterprises eliminated by developed cities. Additionally, these enterprises are generally characterized by high emissions and high pollution. At the same time, the county-level environmental governance constraints are relatively weak, and enterprises have not paid enough attention to the pollution discharge in the production process. The Xiangshui explosion accident on 21 March 2019 is an example. Therefore, counties that concentrate a large proportion of China’s carbon emissions should be the main battlefield for China’s carbon reduction and decarbonization. Thus, can counties achieve carbon emission reduction while pursuing economic growth, that is, whether carbon emissions decouple from economic growth? What factors affect the relationship between the two? If there are no decoupling, how to balance the two? To answer these questions, in-depth research on carbon emissions at the county level is urgently needed.

In addition, China has a vast territory, and there are obvious differences in county development. One-size-fits-all emission reduction policies and measures are less effective. Therefore, it is necessary to carry out a specific and in-depth analysis of carbon emissions at the county level of specific provinces. Jiangsu is the most representative province. At present, Jiangsu has entered the stage of post-industrialization and its GDP per capita reached the level of “middle-upper” developed countries. At the same time, Jiangsu’s carbon emissions have continued to rise, ranking third among all provinces in China. Moreover, there is a large gap in the development level of Jiangsu counties. The county economy in Southern Jiangsu is extremely developed, while Northern Jiangsu lags. Considering the dynamic relationship between carbon emissions and economic growth in Jiangsu counties with different economic development levels, it has important reference significance for other provinces in China, especially those with uneven economic development levels. Therefore, this paper chooses Jiangsu province as an example to examine the decoupling relationship between carbon emissions and economic growth in China’s counties.

The relationship between carbon emissions and economic growth has always been a hot topic in academic research. In the context of global warming [10], the decoupling of carbon emissions and economic growth is the focus of current research. Throughout the relevant literature, the decoupling analysis and environmental Kuznets curve hypothesis are two useful analysis tools. The former describes the correlation between economic growth and carbon emissions from a short-term and real-time perspective [4,11], which is a speed decoupling analysis. The latter assumes that in the early stage of economic development, pollution emissions increase with the level of economic development, and, after crossing the turning point, pollution emissions decrease with the level of economic development [12,13]. It depicts the long-term relationship between carbon emissions and economic growth, and cannot capture their interactions in specific years or stages [4,11], which is a quantitative decoupling analysis. The two methods have their own emphasis and complement each other. Therefore, this paper uses decoupling analysis and environmental Kuznets curve to carry out the analysis.

The structure of this paper is as follows: Section 1 is the introduction; Section 2 is the literature review; the models, data and study area are introduced in Section 3; empirical results and discussion are reported in Section 4; and the conclusion and enlightenment are presented in Section 5.

## 2. Literature Review

### 2.1. Decoupling Analysis

The concept of decoupling, which originated in the field of physics, refers to the disappearance of the correlation between two or more physical variables that are related to each other. Later, it was widely used in the fields of environment–economy to reveal the relationship between economic development and environmental pressure. The OECD decoupling index model proposed by the Organization for Economic Cooperation and Development in 2002 [14] and the elasticity decoupling index constructed by Tapio in 2005 [15] are two decoupling analysis models in existing studies.

#### 2.1.1. OECD Decoupling Index Analysis

A few scholars used the OECD decoupling index model. For example, Mulder et al. investigated the decoupling of energy consumption and economic growth in 10 manufacturing sectors in 14 OECD countries [16]. De Freitas et al. examined the decoupling of Brazil’s economic activity, carbon dioxide emissions and energy consumption [17]; Roinioti et al. explored the decoupling status of Greece’s carbon dioxide emissions and economic growth [18]. Yu et al. analyzed the decoupling relationship between the discharge of soot, chemical oxygen demand and ammonia nitrogen and economic growth in China [19]. The empirical results varied by country, for example, Greece’s economic activity did not decouple with carbon emissions, while China’s pollution emissions completely decoupled from economic growth.

#### 2.1.2. Tapio Elasticity Analysis

Because the OECD decoupling index model is simplistic, previous related studies were mostly conducted with the Tapio elasticity model. In terms of its content, some literatures only focused on the degree of decoupling and its evolution. For example, Zhang et al. [20] and Wang et al. [21] discussed the decoupling relationship between China’s energy-related carbon dioxide emissions and economic growth, and its changes. There are also studies for multiple countries, such as the analysis of China and the U.S.A. [22], China and ASEAN countries [23], 133 countries in the world (with different income levels) [24], and 15 countries (developed and developing countries) [25]. The findings varied from country to country, showing the characteristics of income heterogeneity and period stage. In addition to country-level analysis, there are also studies on provinces, regions and typical regions, such as the analysis of the decoupling of economic growth and carbon dioxide emissions in China’s provinces and Beijing–Tianjin–Hebei regions [10,26], and the research on the decoupling relationship between carbon dioxide emissions of the commercial building sector and economic growth of the tertiary industry in four municipalities in China [27]. Wang et al. [28] and Wang et al. [29] examined the decoupling status of carbon dioxide emissions from the transportation sector in China’s Jiangsu province and the power sector in Shandong province, while Chen et al. evaluated the coupling relationship between energy use and carbon emissions in Beijing and Issaquah cities [30]. There are significant differences in the conclusions of these empirical studies.

Some literatures not only investigated the decoupling relationship between the two, but also explored its driving factors. The measurement and factor decomposition analysis of the decoupling effect of carbon dioxide emissions were carried out for the United States [31], OECD [32], the BRICS countries [33] and Germany, Japan, South Korea and China Taiwan [34]. The first three focused on the overall economy, and the fourth was based on the road transportation industry. In addition, there are also Chinese studies. Zhao et al. [35] and Yang et al. [36] examined the decoupling status and its driving factors of China’s economic growth and carbon dioxide emissions from the perspective of industry. Zhou et al. selected eight regions in China for research [37]. There are also discussions on individual industry, such as the analysis of the decoupling effect and its cause of carbon dioxide emissions in China’s provincial construction industry [38] and agriculture [39].

#### 2.1.3. Combination of the OECD Decoupling Index and Tapio Elasticity Model

Few studies used both the OECD decoupling index and Tapio elasticity model. For example, Lin et al. adopted two methods to evaluate the decoupling effect of South Africa’s GDP and carbon emissions, and found that there was strong decoupling only from 2010–2012 [40]. Wu et al. analyzed typical developed and developing countries, and the results showed that developed countries emerged strong decoupling and slightly improved stability, while developing countries presented weak decoupling with large fluctuations and a lack of regularity [41].

### 2.2. Environmental Kuznets Curve Hypothesis

Since Grossman et al. proposed the Environmental Kuznets Curve (EKC) hypothesis in 1995 [12], a large number of studies have sprung up around it in academia. With the continuous deepening of empirical analysis, its conclusions are also diversified. To sum up, there are three situations.

#### 2.2.1. Supporting the EKC Hypothesis

Some studies confirmed the EKC hypothesis. Among them, some literatures focused on a particular country or region, such as the test of the EKC hypothesis on carbon dioxide emissions in the United States [42,43], the United Kingdom [13], Cunha [44], Pakistan [45,46] and China [47,48]. Additionally, the conclusions unanimously supported the EKC hypothesis. In addition, the conclusions are the same for some studies focusing on the world or certain regions. For example, the test results for Australia, China, Canada and the U.S.A. [49], BRIC countries [50,51], some Middle Eastern and North African countries [52,53], 14 Asian countries [54], 10 sub-Saharan African countries [55] and 124 countries [56] also confirmed the validity of the EKC hypothesis. In addition to carbon dioxide emissions, there are also analyses of the EKC effects of other pollutants. For example, the test of urban solid waste in India also provided empirical support for the existence of the EKC hypothesis [57].

#### 2.2.2. Opposing the EKC Hypothesis

Some studies believed that the EKC effect did not exist. For example, Turkey’s real GDP per capita significantly inhibited carbon dioxide emissions per capita [58], there was no clear evidence to show the inverted U-shaped relationship between economic growth and carbon dioxide emissions in West Africa [59,60], and the growth of GDP in five North African countries led to an increase in carbon dioxide emissions [61]; all these results disagreed with the EKC hypothesis. There is also evidence from other pollutants. For example, the empirical analysis of NOx and sulfur dioxide emissions in the Netherlands, the United Kingdom, the United States and West Germany [62], sulfur emissions in 74 countries [63] and in global developed and developing countries [64] also confirmed that the EKC hypothesis was not valid.

#### 2.2.3. Neither Supporting nor Opposing the EKC Hypothesis

Other studies suggested that the validity of the EKC hypothesis was uncertain. On the one hand, the uncertainty of the conclusions was manifested in different pollutants. For example, Fodha et al. found that Tunisia’s sulfur dioxide emissions per capita had an inverted U-shaped relationship with the GDP per capita, while carbon dioxide emissions per capita were monotonically linear [65]. Brajer et al. believed that the pollutant measurement methods in China were different, and the EKC conclusions were also different [66]. The uncertainty of conclusions, on the other hand, was reflected in the differences in the scope of the study (e.g., whole versus local or individual). For example, Olale et al. believed that the EKC hypothesis of carbon emissions in Canada was valid for the entire country, but the provinces/regions were the opposite [67]. Jaunky found there was a positive linear relationship between real GDP per capita and carbon dioxide emissions per capita in 36 high-income countries, but an individual analysis of Greece, Malta, Oman, Portugal, and the United Kingdom supported the EKC hypothesis [68]. Alam et al. believed that India’s income was positively correlated with carbon dioxide, while Indonesia, China and Brazil showed an inverted U-shaped relationship [69]. Qiao et al. found that the EKC effect existed in the whole sample and developed economies, while was positively correlated in developing economies [70]. However, Stern et al. believed that sulfur emissions per capita was a monotonous linear function of income per capita globally, but it was an inverted U-shaped function of income in high-income countries [71]. Similarly, empirical analyses of the applicability of the carbon emission EKC in different income countries produced mixed conclusions. For example, Ogundipe et al. supported the establishment of the EKC hypothesis only in a low-income group in Africa [72], Azam et al. recognized the existence of the EKC effect only in low-income and low-middle-income countries [73], and Tachega et al. found that the EKC hypothesis was effective only in the samples of low-income, middle-income and upper-middle income economies in Africa [74]. There is also empirical evidence from different industries. For example, the EKC effect did not exist in the agricultural sector of all 4 income groups in 115 countries, but existed in the manufacturing sector [75].

### 2.3. Literature Commentary

Overall, the decoupling theory and the EKC hypothesis are two powerful tools to analyze the relationship between carbon emissions and economic growth. Scholars used them to investigate the dynamic relationship between global, national, or intra-national regional carbon emissions and economic growth. Various conclusions were obtained, such as the decoupling status changed with the research scope and research period. Similarly, the EKC conclusions were mixed. Previous studies have been relatively mature, and achieved more results. Compared with the OECD decoupling index model, the Tapio elasticity model is more comprehensive. However, there are still three shortcomings. First, there are many relevant analyses involving China’s carbon emissions, but they all focus on China or provinces (regions) [4,19,20,21,26,27,28,29,35,36,37,38,39,47,66], lacking discussions at the county level. The county area, as the basic strategic unit for the transformation of China’s current and future economic growth mode, is precisely the focus of China’s carbon reduction and decarbonization in the future. Second, the existing studies believe that the reasons for the inconsistent EKC test results lie in the research samples, selection of variables and measurement methods, and few literatures mention income differences. However, empirical evidence shows that the heterogeneity of income should be an important factor that cannot be ignored [70,71,72,73,74]. In addition, the 2008, the global economic crisis changed the evolution pattern of carbon emissions in many countries, including China [76]. However, the current EKC research does not mention the impact of the crisis. Third, most studies either adopt the decoupling theory or choose the EKC hypothesis to analyze the dynamic relationship between carbon emissions and economic growth from the perspective of speed or quantity. Only a few studies adopted both of them [4,11]. In fact, they have their own advantages and should complement each other. In view of this, this paper combines the Tapio elasticity model and the EKC hypothesis to empirically examine the relationship between carbon dioxide emissions and economic growth in Jiangsu counties, and pays attention to income heterogeneity and the impact of the 2008 global economic crisis on the carbon emission EKC. The research aims to explore the carbon reduction and decarbonization strategies of Jiangsu and other provinces in China (different stages of economic development), and then provide directions for the realization of China’s double-carbon goal.

Compared with the previous studies, this paper has three contributions. First, to our knowledge, this is the first study to explore the dynamic relationship between carbon emissions and economic growth at the county level in China, and its conclusions can provide a reference for county low-carbon transformation. Second, the Tapio elasticity model and the EKC model are used for systematic and comprehensive analysis. Third, when testing the EKC hypothesis of county-level carbon emissions, the influences of the heterogeneity of regional economic development levels and the 2008 global economic crisis are considered, so the estimation results are more accurate.

## 3. Models, Data and Study Area

### 3.1. Tapio Elasticity Model

The Tapio elasticity model adopts the “elasticity concept” to dynamically measure the relationship between economic growth and carbon emissions, that is, to illustrate whether there is a synchronous relationship between the two through the “relative ratio of carbon emissions growth rate and economic growth rate”. The specific formula is:

(1) Tit=ΔCitCi0ΔGitGi0=(Cit−Ci0)Ci0(Git−Gi0)Gi0

where $T_{i}^{t}$ is the decoupling elasticity index of carbon emissions and economic growth at a given period from base year 0 to target year t for region i; $C_{i}^{t}$ and $C_{i}^{0}$ are the total carbon emissions in year t and base year 0; and $G_{i}^{t}$ and $G_{i}^{0}$ are the total Gross Domestic Product (GDP) in year t and base year 0. $\Delta C_{i}^{t}$ and $\Delta G_{i}^{t}$ are the increment of carbon emissions and GDP in a given period for region i.

The Tapio elasticity model determines the strength of the decoupling degree of the two according to the elasticity coefficient, which is divided into three statuses: negative decoupling, decoupling and coupling. Among them, negative decoupling is further divided into weak negative decoupling, strong negative decoupling, expansive negative decoupling and recessive decoupling; decoupling is divided into strong decoupling and weak decoupling; and coupling is divided into recessive coupling and expansive coupling. See Table 1 for details.

### 3.2. Carbon Emission EKC Model

The EKC shows that there is an inverted U-shaped relationship between economic growth and environmental pollution. When testing the validity of the carbon dioxide emissions EKC hypothesis, there are three cases for the measurement of variables: carbon dioxide emissions and GDP [50,51,64], carbon dioxide emissions and GDP per capita [46,60,72], and carbon dioxide emissions per capita and GDP per capita [13,30,42,43,44,47,52,53,55,56,61,62,65,66,68,70,73,74]. Referring to the practice of most scholars, this paper selected per capita indicators to test the EKC effect of county-level carbon emissions in Jiangsu province.

Meanwhile, previous studies have shown that in addition to economic activity, industrial structure [3,16,17,49,62,77,78,79], level of opening-up [44,45,47,53,73,77] and energy utilization efficiency (energy intensity) [3,18,20,22,23,26,27,29,33,35,36,47,77,78,79] are also important factors affecting carbon dioxide emissions or their decoupling status from economy growth. In addition, environmental regulation is also an important variable affecting pollutant emission [80,81,82,83]. To avoid estimation bias, the above four variables are introduced into the model as control variables. The industrial structure is measured by the share of the added value of the tertiary industry in GDP. Compared with the primary and secondary industries, the tertiary industry is relatively “cleaner” and consumes less fossil energy per unit output, resulting in relatively low carbon emissions. Therefore, the impact of the tertiary industry’s proportion is expected to be negative. The level of opening-up is measured by the proportion of total import and export in GDP, that is, foreign trade dependence. The “Pollution Heaven Hypothesis” holds that enterprises in pollution-intensive industries tend to be established in countries or regions with relatively low environmental standards [84], so the impact of the level of opening-up in China is expected to be positive. For energy utilization efficiency, since the county energy data is limited, industrial power utilization efficiency (i.e., the reciprocal of industrial power consumption intensity) is selected as a proxy variable. Energy utilization efficiency is expected to have a negative impact, because the higher the energy utilization efficiency, the lower the energy consumption unit output and the lower the carbon emissions, correspondingly. Environmental regulation is measured by the proportion of fiscal environmental protection expenditure in the GDP. The direction of its expected impact on carbon emissions is uncertain. In theory, environmental regulations not only directly affect carbon emissions, but also may affect carbon emissions through intermediate variables, such as technological progress and the energy consumption structure [83]. Additionally, the mechanism is complex. For example, the impact of environmental regulation on the level of green technology is non-linear. When the level of regulation is low, it inhibits the progress of green technology and leads to the increase in carbon emissions. When the regulation level reaches a certain level, enterprises are encouraged to carry out green innovation activities and promote the progress of green technology, thereby curbing the increase in carbon emissions [85]. Moreover, empirical analysis confirmed that the impact of environmental regulation on carbon emissions has three conditions: promotion [80], inhibition [81] and uncertainty [82].

In addition, considering that the 2008 global economic crisis may change the relationship between economic growth and carbon emissions, a dummy variable $D_{it}$ is introduced into the model. Specifically, the year 2008 is selected as the node, and the post-crisis period is the dummy variable, that is, if the year is after 2008, take 1, otherwise it is set to 0. At the same time, since this paper focuses on the relationship between economic growth and carbon emissions, it is necessary to introduce the interaction terms between the dummy variable and economic growth variables ($Dit*r_{Git}$ and $Dit*r_{Git}^{2}$). Ultimately, the model constructed in this paper is as follows:

(2) $$r_{Cit}=\gamma_{0}+\gamma_{1}D_{it}+\beta_{11}r_{Git}+\beta_{21}r_{Git}^{2}+\beta_{12}D_{it}*r_{Git}+\beta_{22}D_{it}*r_{Git}^{2}+\alpha_{1}S_{it}+\alpha_{2}F_{it}+\alpha_{3}E_{it}+\alpha_{4}Er_{it}+\varepsilon_{it}$$

where i represents the county and t represents the year. $r_{C}$ and $r_{G}$ represent the carbon emissions per capita and GDP per capita, respectively; S, F, E and Er denote the industrial structure, level of opening-up, energy utilization efficiency and environment regulation, respectively. $\gamma_{0}$, $\gamma_{1}$, $\beta_{11}$, $\beta_{21}$, $\beta_{12}$, $\beta_{22}$, $\alpha_{1}$, $\alpha_{2}$, $\alpha_{3}$ and $\alpha_{4}$ are the parameters to be estimated; $\varepsilon_{it}$ is the random disturbance term. Whether the carbon emission EKC holds up depends on $\beta_{21}$ and/or $\beta_{22}$. If $\beta_{21}<0$ and/or $(\beta_{21}+\beta_{22})<0$ pass/passes the significance test, it means that the two have an inverted U-shaped relationship, that is, the EKC is established.

Meanwhile, if at least one of $\gamma_{1}$, $\beta_{12}$ and $\beta_{22}$ is significantly different from 0, it indicates that the 2008 global crisis changed the relationship between economic growth and carbon emissions. Among them, only when $\gamma_{1}$ is significantly different from 0, the turning point of the curve does not change, and only when $\beta_{12}$ and/or $\beta_{22}$ are/is significantly not 0, the turning point changes.

### 3.3. Study Area and Data

#### 3.3.1. Study Area

The county-level units in this paper include three types of administrative units: municipal districts, counties, and county-level cities. Jiangsu province is located in the Yangtze River Delta region where the adjustment of administrative divisions is relatively active, so it is necessary to carefully select the county samples. Using Jiangsu administrative division in 2010 as the standard, the counties that were adjusted (for example, removing counties into districts and dismantling counties into cities) during the sample period are eliminated and merged, and 53 counties are established as the analysis sample. Among them, there are 14 counties in Southern Jiangsu, 14 counties in Central Jiangsu, and 25 counties in Northern Jiangsu (see Table A1 for details). Meanwhile, due to the availability of data, the research period of this paper is 2000–2017.

#### 3.3.2. Data Source and Description Analysis

The county carbon emission data is obtained from http://www.ceads.net.cn (18 February 2022). The county-level carbon emission data provided by the CEADs database (18 February 2022) is more accurate, because it uses the particle swarm optimization-back propagation (PSO-BP) algorithm to unify the two sets of nighttime light data of DMSP/OLS and NPP/WIIRS [86]. Unfortunately, the current data of carbon emissions is only updated to 2017. The data of the county GDP, GDP per capita, tertiary industry added value, industrial added value, industrial electricity consumption, total population and foreign trade dependence are from the Jiangsu Statistical Yearbook and its prefecture-level city Statistical Yearbook over the years. The county-level environmental protection fiscal expenditure is estimated based on the prefecture-level city’s environmental protection fiscal expenditure and its share of industrial added value in the prefecture-level city. Among them, the data of prefecture-level city environmental protection fiscal expenditure are from the corresponding prefecture-level city Statistical Yearbook. In order to eliminate the impact of price factors, the GDP per capita and industrial added value are reduced at constant prices in 2000. Missing data in individual years are filled with the mean of the two adjacent groups. 

The descriptive statistical analysis of the data is shown in Table A2. It shows that the development of counties in Jiangsu is significantly different. The level of economic development is decreasing in the order of Southern Jiangsu–Central Jiangsu–Northern Jiangsu, and Central and Northern Jiangsu are relatively close. For example, during the study period, the average GDP per capita $\left(r_{G}\right)$ of the three regions was 64,300 CNY/person, 30,000 CNY/person, and 19,800 CNY/person, respectively. The same is true for the carbon emissions per capita $\left(r_{C}\right)$, the proportion of the tertiary industry (S), foreign trade dependence (F) and energy utilization efficiency (E). In particular, the level of foreign trade dependence is “far ahead” in Southern Jiangsu. However, the ranking of environmental regulation (Er) in inter-regions has changed, but it is still the highest in Southern Jiangsu, and the lowest in Northern and Central Jiangsu. These characteristics can be observed from the means of the data in the three regions.

## 4. Results and Discussion

### 4.1. The Tapio Elasticity Analysis

Combined with the relevant carbon emissions and constant price GDP data, calculate the county Tapio elasticity coefficients by using Formula (1), and clarify whether its carbon emissions are decoupling from economic growth.

#### 4.1.1. Overall Analysis

**Figure 1:**
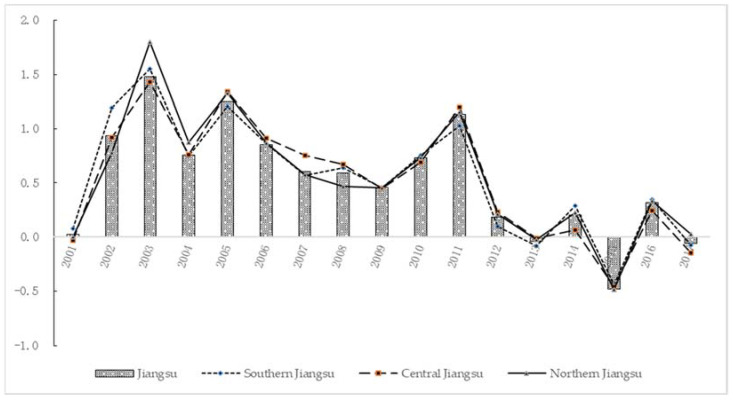
Tapio elasticity coefficients of Jiangsu and three regions (2001–2017).

The results show that from 2001 to 2017, the carbon emissions and economic growth in Jiangsu counties experience four decoupling statuses: weak decoupling (2001, 2004, 2007–2010, 2012, 2014 and 2016), expansive coupling (2002, 2006 and 2011), expansive negative decoupling (2003 and 2005) and strong decoupling (2013, 2015 and 2017). This also indicates that since 2012, the weak decoupling and strong decoupling effects of carbon emissions in Jiangsu counties have appeared alternately. From a regional perspective, the decoupling effect of carbon emissions in Southern Jiangsu is the same as that in Jiangsu, merely the degree is different. The same is true for Northern Jiangsu and Central Jiangsu except for certain years (Central Jiangsu 2001 and 2011, Northern Jiangsu 2004 and 2017). In addition, the calculation results show that the evolution of the Tapio elasticity index in Jiangsu and the three regions has changed a lot around 2008. In other words, the 2008 global economic crisis may have changed the relationship between Jiangsu’s economic growth and carbon emissions. See Figure 1 for details (see Table A3 for data).

**Figure 2:**
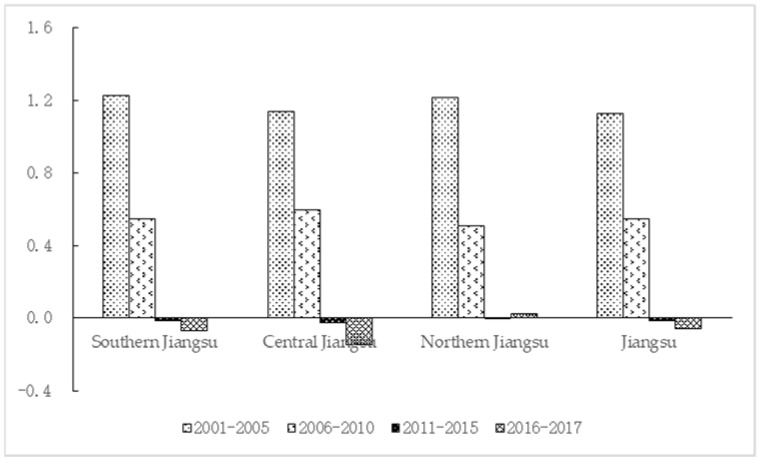
Tapio elasticity coefficients of Jiangsu and three regions (by time period).

Viewed by time periods (the time division in line with China’s five-year plan), the decoupling effect of carbon emissions in Jiangsu counties gradually strengthened. Specifically, the carbon emissions decoupling elasticity coefficients in 2001–2005, 2006–2010, 2011–2015 and 2016–2017 were 1.126, 0.546, −0.015 and −0.060, corresponding to expansive coupling, weak decoupling, strong decoupling, and strong decoupling, respectively. This shows that Jiangsu’s carbon emission reduction effect is obvious, especially since 2011. However, in recent years, the situation in Northern Jiangsu has been slightly worse. In 2016–2017, carbon emissions and economic growth still remains in a weak decoupling status, and the decoupling effect is weakened. See Figure 2 for details (see Table A4 for data).

#### 4.1.2. Individual Analysis

Table 2 shows that from 2001 to 2005, carbon emissions and economic growth in 30 counties were in a status of expansive coupling (II). Among them, there were 6 in Southern Jiangsu, 9 in Central Jiangsu, and 15 in Northern Jiangsu. A total of 21 counties showed expansive negative decoupling (I), including 6 in Southern Jiangsu, 5 in Central Jiangsu, and 10 in Northern Jiangsu. Two counties showed weak decoupling (III), both of which belong to Southern Jiangsu. From 2006 to 2010, 50 counties showed weak decoupling (III), and the remaining 3 counties showed expansive coupling (II), including 1 in Southern Jiangsu, 1 in Central Jiangsu and 1 in Northern Jiangsu. From 2011 to 2015, there were 25 counties with weak decoupling (III) in their carbon emissions, including 9 in Southern Jiangsu, 6 in Central Jiangsu, and 10 in Northern Jiangsu. The remaining 28 counties showed the characteristics of strong decoupling (IV), including 5 in Southern Jiangsu, 8 in Central Jiangsu, and 15 in Northern Jiangsu. From 2016 to 2017, 23 counties (8 in Southern Jiangsu, 2 in Central Jiangsu and 13 in Northern Jiangsu) and 30 counties (6 in Southern Jiangsu, 12 in Central Jiangsu and 12 in Northern Jiangsu) showed weak decoupling (III) and strong decoupling (IV) characteristics. It can be seen that since 2011, 53 counties in Jiangsu achieved the decoupling of carbon emissions and economic growth. In particular, the counties with strong decoupling characteristics are distributed in Southern, Central or Northern Jiangsu, which also indicates that whether economic growth decouples from carbon emissions is not necessarily related to geographical division. However, Central Jiangsu has the highest proportion of counties that entered a strong decoupling stage, which is different from Tapio’s [15] research conclusion of a higher proportion of counties entering a decoupling status for high-income groups. To some extent, this indicates that the county economy in Jiangsu has been an extensive rather than intensive development mode in the past few decades. 

However, 47.17% of the counties (25 counties) experienced different degrees of decline in the decoupling effect of carbon emissions in recent years. Comparing the Tapio elasticity coefficients in 2011–2015 and 2016–2017, it is found that those of 25 counties increased, indicating that the decoupling effect of carbon emissions in these areas weakened. Among them, 8 are located in Southern Jiangsu, 2 in Central Jiangsu, and 15 in Northern Jiangsu. Especially for Gaochun, Liyang, Yangzhong, Jurong (Southern Jiangsu); Jingjiang, Jiangyan (Central Jiangsu); Pizhou, Lianshui and Suyu (Northern Jiangsu), the decoupling effect declined more, which can be seen from the difference of the Tapio elasticity coefficients between the two phases, which is greater than 0.30. Therefore, great attention should be paid to the fact that the decoupling effect of carbon emissions in nearly half of Jiangsu counties has weakened in recent years.

In summary, Jiangsu counties experienced four decoupling statuses of carbon emissions and economic growth during 2000–2017. Since 2011, all counties achieved a decoupling of the two, with more than half of them showing strong decoupling feature. Additionally, the proportion of counties with strong decoupling in Central Jiangsu was relatively high. It can be seen that Jiangsu counties achieved the speed decoupling of carbon emissions and economic growth, and the intensity of decoupling was not positively correlated with the level of regional economic development.

### 4.2. The EKC Analysis

#### 4.2.1. Full-Sample Analysis

The panel unit root test. The same order single integration of variables is the prerequisite of cointegration analysis, and the unit root test is usually used to identify the single integer order of variables. To date, the LLC test, Breitung test, IPS test, ADF Fisher test, and PP Fisher test are common unit root tests for panel data. Among them, the first two are suitable for the same root sequence, and the last three are suitable for different root sequences. The unit root tests are carried out on 7 series, including carbon emissions per capita in Jiangsu counties, and the results are shown in Table A5.

As shown in Table A5, the results of the LLC test, Breitung test, IPS test, ADF Fisher test and PP Fisher test of $r_{C}$ all accept the null hypothesis, and the first-order difference rejects the null hypothesis. According to the criterion of the unit root test, it can be known that $r_{C}$ is a non-stationary series and its first-order difference is a stationary series. In other words, it is an integral sequence of first order. Similarly, the same is true for the other 6 variables (except for the Breitung test conclusion of $r_{G}^{2}$). It can be seen that all of these variables are first-order single-integration series, which can be used for panel cointegration analysis.

The panel cointegration test. Then, the Pedroni test, Kao test and Fisher test are used to examine whether there is a cointegration relationship between the panel variables. The results are shown in Table A6. It shows that the five statistics of the Pedroni test significantly reject the null hypothesis that there is no cointegration. The Kao test and Johansen Fisher test also draw the same conclusion. Therefore, it can be concluded that there is a cointegration relationship among the 7 variables. Further, Equation (2) is estimated. The mixed WLS, fixed-effects and random-effects models were estimated in turn, as shown in Table 3. 

Meanwhile, the F test and Hausman test for the selection of the three types of models were listed. The results show that the fixed-effects model is more suitable than the mixed WLS model, because the F test significantly rejects the null hypothesis of the mixed WLS model. Similarly, the Hausman test finds that the null hypothesis of the random-effects model is rejected, so the fixed-effects model is also better than the random-effects model. Therefore, the fixed-effects model was selected for analysis, and the mixed WLS model and random-effects models were only used for reference.

The curve fitting effect of variables, such as the carbon emissions per capita $\left(r_{C}\right)$ and GDP per capita $\left(r_{G}\right)$, in Jiangsu counties is very good, and the value of the adjusted R-squared is not less than 0.90. Moreover, the F test shows that the equation passed the joint test. The three regression equations all have $\beta_{11}>0$, $\beta_{21}<0$, $\beta_{12}<0$ and $\beta_{22}<0$, and passed the significance test of 1% or 5%. Therefore, the EKC hypothesis of county carbon emissions in Jiangsu is established, and the 2008 global economic crisis really changed the shape of the carbon emission EKC. The dummy variables (D) of the three types of regression models are set to 0 and 1 in turn, and the EKC equations for 2000–2008 and 2009–2017 are obtained. Further, the turning points of the curves in the corresponding periods are calculated, and the turning points of the latter period are listed in Table 3. 

The results show that, taking the constant price in 2000 as the benchmark, the turning point of county carbon emissions per capita in the fixed-effects model occur when the GDP per capita is CNY 132,478. Additionally, the GDP per capita corresponding to the turning points of the mixed WLS model and the random-effects model are CNY 125,895 and CNY 145,095, respectively. Compared to the data ($r_{G}$), the GDP per capita of Zhangjiagang, Jiangyin, Taicang, Kunshan and Changshu, which ranked as the top five in 2017, reached more than CNY 140,000. This means that they were all on the right side of the carbon emission EKC, while the remaining 48 counties did not reach or exceeded the turning point and were on the left side of the curve. That is, from a quantity perspective, to date, only Zhangjiagang, Jiangyin, Taicang, Kunshan and Changshu achieved the decoupling of carbon emissions and economic growth.

For the fixed-effects model, the industrial structure (S), foreign trade dependence (F) and energy utilization efficiency (E) all pass the 1% significance test. Among them, the coefficient of foreign trade dependence is greater than 0, the coefficients of the industrial structure and energy utilization efficiency are less than 0, and the estimated symbols of the coefficients are consistent with theoretical expectations. The level of opening-up positively promoted the increase in the regional carbon emissions per capita. This is consistent with the findings of Al-Mulali et al. [44], Nasir et al. [45], Jalil et al. [47], Farhani et al. [53], and Azam et al. [73], who believed that trade openness had a positive impact on (or positive correlation with) carbon emissions (or environmental degradation and air pollution). The increase in the proportion of the tertiary industry inhibited the increase in carbon emissions per capita, which shows that the adjustment of industrial structure is conducive to reducing carbon emissions. This is in line with the ideas that suggest that “structural change greatly promoted the growth of manufacturing energy productivity and led to the decline of carbon emissions (Mulder et al.) [16]”; “economic structure adjustment was related to carbon emission reduction (De Freitas et al.) [17]”; “The decline of carbon emissions in developed countries was attributed to structural change (Sarkodie et al.) [49]”; and “emissions reduction was the result of economic structural change (De Bruyn et al.) [62]”. Similar to industrial structure, energy utilization efficiency is also a restraining effect, which indicates that improving energy efficiency, that is, reducing energy intensity, helps to hinder the increase in carbon emissions. This conclusion is validated in quite a few studies [18,20,22,23,26,27,29,33,35,36,47]. The environmental regulation (Er) coefficient is greater than 0, but fails the significance test, which is consistent with the findings of Zhang et al. [81]. This indicates that during the sample period, the positive effect of environmental regulation in Jiangsu counties on its carbon emissions per capita is not obvious. The possible reason is that, on the whole, the level of environmental regulation was relatively low, and the lower regulation cost did not affect the “existing extensive production and operation mode” of enterprises, which, in turn, led to an increase in carbon emissions, but this effect is not obvious.

#### 4.2.2. Different Income Groups Analysis

The full-sample analysis shows that currently only 5 counties in Southern Jiangsu achieved the decoupling of carbon emissions and economic growth, which is significantly different from the conclusion of the Tapio elasticity analysis. The reason may be that the differences in the regional economic development levels were not considered when examining the EKC effect of county carbon emissions. However, related studies showed that income heterogeneity was also an important factor affecting the establishment of the EKC [70,71,72,73,74].

The level of economic development in Jiangsu counties shows a gradual decline in Southern Jiangsu—Central Jiangsu—Northern Jiangsu. In general, Central Jiangsu is closer to Northern Jiangsu, but significantly lower than Southern Jiangsu (See Section 3.3.2 for details). Therefore, 14 counties in Southern Jiangsu are regarded as a high-income group”, and the remaining 39 counties in Central and Northern Jiangsu are regarded as a “low-income group” to test the effectiveness of the carbon emission EKC of different income groups. 

Similar to the full-sample analysis, the EKC equations of carbon emissions in the high-and low-income groups are estimated in turn. For the high-income group equation, the coefficient of the interaction term $\left(D*r_{G}\right)$ is not significant, so it is not included in the model. Table 4 lists the estimation results and model selection test statistics of the two. The F test and Hausman test show that the low- and high-income groups are suitable for the fixed-effects model.

As can be seen from Table 4, the county carbon emission EKCs of both low- income and high-income groups are established. Additionally, the carbon emission EKCs of the two groups changed significantly before and after the 2008 global economic crisis. Similar to the full-sample analysis, the turning points of the curve are calculated between 2009–2017. The results indicate that the level of GDP per capita at the turning point of carbon emissions during this period varies greatly, which is CNY 74,618 and CNY 143,387, respectively. Furthermore, as far as the low-income group is concerned, Haimen, Qidong, Tongzhou, Dafeng, and Jingjiang, of which all GDP per capita exceeded the turning point, were on the right side of the EKC in 2017. With the increase in the GDP per capita in these counties, the carbon emissions per capita did not increase, but decreased. In other words, the decoupling of carbon emissions and economic growth was achieved. The other 34 counties did the opposite. For the high-income group, Zhangjiagang, Jiangyin, Taicang, Kunshan and Changshu were the counties for which the GDP per capita exceeded the turning point in 2017, which is consistent with the conclusion of the full sample analysis. 

The impacts of the four control variables on the carbon emissions are different for the high- and low-income groups. Among them, the coefficients of the tertiary industry’s share and energy utilization efficiency are significantly less than 0, and the coefficients of the low-income group are larger. This indicates that the inhibitory effect of industrial structure and energy utilization efficiency on carbon emissions per capita in the high-income group is stronger than that in the low-income group. The possible reason is that, compared to Northern and Central Jiangsu, the tertiary industry in Southern Jiangsu accounts for a higher proportion, as well as the energy utilization efficiency, so they have a stronger hindering effect on the increase in carbon emissions. The foreign trade dependence coefficient is greater than 0, but only the high-income group passes the significance test. This shows that it has no significant impact on the carbon emissions per capita of counties in the low-income group, while the high-income group plays a significant positive role. This is consistent with the level of regional opening-up. The opening-up level of Central and Northern Jiangsu is low, so the “Pollution Heaven effect” has not yet appeared, but the Southern Jiangsu region is the opposite. The environmental regulation coefficient of the low-income group is significantly greater than 0, and that of the high-income group is less than 0 and insignificant. It means that the environmental regulation in Northern and Central Jiangsu promoted the increase in carbon emissions, while the environmental regulation in Southern Jiangsu had an insignificant hindering effect. This is consistent with the conclusions of Wang et al. and Yang et al. Wang et al. that supported the inverted U-shaped relationship between carbon emissions and environmental regulations [82]. When the regulation level is low, it plays a positive promoting effect; when exceeding a certain level, it turns into a negative inhibitory effect. This conclusion is applied to this paper, which also concludes that Central and Northern Jiangsu are the promoting effects, and Southern Jiangsu is the hindering effect. Additionally, Wang et al. found that the direct effect of environmental regulation was positive, and the indirect effect was negative, and the lower the level of regulation, the smaller the indirect effect [83]. Therefore, for Central and Northern Jiangsu with lower intensities, the direct effect dominated, and so it is reflected as a positive promoting effect. Sothern Jiangsu is the opposite, but the hindering effect was not significant. 

In conclusion, the difference in the regional economic development levels should be considered when testing the EKC effect. The EKC of county carbon emissions in different income groups was established, and the curve changed before and after the 2008 global crisis. In 2017, only 10 of the 53 counties were on the right side of the curve, achieving the quantitative decoupling between economic growth and carbon emissions. Industrial structure and energy utilization efficiency hindered the increase in county carbon emissions. The impacts of the opening-up level and environmental regulation were different for low-income and high-income groups.

## 5. Conclusions and Implications

### 5.1. Conclusions

Based on the panel data of Jiangsu counties from 2000 to 2017, this paper empirically examines the relationship between carbon emissions and economic growth from the dual perspective of speed decoupling and quantity decoupling. We used the Tapio elasticity model and the EKC model, and pay attention to the impacts of income heterogeneity and the 2008 global economic crisis. The main conclusions are as follows.

During the study period, the relationship of Jiangsu county carbon emissions and economic growth experienced four statuses: weak decoupling, expansive coupling, expansive negative decoupling, and strong decoupling. In terms of the different periods, the decoupling effect of carbon emissions gradually increased, especially since 2011, and all counties achieved the decoupling. Among them, more than half showed a strong decoupling, and the proportion of counties in Central Jiangsu was relatively high. However, in recent years, the decoupling intensity in 47.17% of the counties’ carbon emissions declined, and 60% of them are in Northern Jiangsu. In summary, Jiangsu counties achieved the speed decoupling of carbon emissions and economic growth.

The result of the full-sample analysis is quite different from the Tapio elasticity result. Therefore, the heterogeneity of regional economic development level should be considered when testing the EKC effect. The relationship between county carbon emissions and economic growth in different income groups is an inverted U shape, and the curve changed before and after the 2008 global economic crisis. In 2017, only 10 counties are on the right side of the curve, achieving the quantitative decoupling between economic growth and carbon emissions; 43 counties are on the left side of the curve and in the “dilemma” between economic growth and carbon emissions reduction. Therefore, the decoupling of carbon emissions and economic growth was not achieved in all counties in Jiangsu.

The industrial structure and energy utilization efficiency significantly hinders the increase in the carbon emissions per capita in Jiangsu counties, especially in the economically developed Southern Jiangsu region. The level of opening-up is a promoting effect, but the effect is not significant in Central and Northern Jiangsu. The influence of environmental regulation is more complex, with Central and Northern Jiangsu as a significant promoting effect, and Southern Jiangsu as an insignificant hindering effect.

### 5.2. Enlightenment

The above research conclusions indicate that the current environmental policies cannot take into account carbon emission reductions and economic development. To achieve a win–win situation between the two, we need to make efforts concerning various aspects, such as industrial structure and energy utilization efficiency. 

First, actively adjust and optimize the industrial structure. The results show that the proportion of the tertiary industry inhibits the increase in carbon emissions per capita, and the impact of the low-income group (Central Jiangsu and Northern Jiangsu) is less than that of the high-income group (Southern Jiangsu). Therefore, vigorously develop the tertiary industry, especially the high-tech service industries, such as financial services and information technology in Northern and Central Jiangsu. Meanwhile, transform traditional industries, and to realize the upgrading of industrial structures. For example, providing preferential policies, financial support and other measures to encourage enterprises to upgrade from low value-added to high value-added products, and from high pollution to low pollution. Strengthen environmental regulations of industries with a high energy consumption, high pollution and high emissions, and force enterprises to solve pollution problems.

Second, strive to improve energy utilization efficiency, and strengthen technological exchanges and cooperation between regions. It is also an effective way to reduce carbon emissions by increasing investments in green research and development, promoting innovation in energy-saving and environmental protection technologies, accelerating the development and utilization of clean energy and renewable energy, and promoting the rational and efficient use of energy to achieve carbon emission reduction. At the same time, low-carbon technology is relatively advanced in developed regions (Southern Jiangsu), and underdeveloped regions (Central Jiangsu and Northern Jiangsu) should strengthen their exchanges and learning with them, and shorten the process of technology research and development, so that effective energy-saving and emission-reduction technologies can be rapidly spread and spillover, and continue to play the role of technology in emission reduction. 

In addition, actively improve the foreign trade structure, especially in Southern Jiangsu. Pay more attention to the introduction of foreign advanced low-carbon technologies, encourage the export of low energy consumption, low pollution and low value-added products, and optimize the import and export trade structure, so as to reverse the carbon emissions promotion effect of foreign trade dependence. Appropriately enhance the intensity of environmental regulation, and urge enterprises to make structural adjustments with more potential technological development models. Further implement environmental protection policies, pay attention to the implementation of various links, and urge enterprises to gradually change their development strategies based on extensive industrial structures.

In short, the implementation of differentiated emission reduction strategies is the first criterion, and structural emission reduction and technological emission reduction are the two main approaches.

First of all, the situation of counties in the Yangtze River Delta is similar to those in Jiangsu counties, so the above conclusions and suggestions can provide references for county carbon emission reduction measures in the counties in the Yangtze River Delta. Additionally, the emission reduction strategies based on the local conditions caused by the unbalanced regional development of Jiangsu also has certain reference values for specific provinces with an unbalanced economic development. Third, compared to the previous macro-level analysis at national and provincial levels (regions), the county-level time-division study in this paper reveals the impact of the 2008 global crisis on the relationship between carbon emissions and economic growth, providing a useful attempt for the more detailed study of carbon emissions.

However, there are still certain limitations in this paper. Due to the lack of county-level data in China, the influencing factors only considered industrial structure, energy utilization efficiency, foreign trade dependence and environmental regulation, and have not yet involved factors, such as green finance and R&D investment. In addition, this paper does not focus on the spatial effect of county carbon emissions. These two points are also the research directions for the future.

## Figures and Tables

**Table 1:** Tapio decoupling status classification.

$T_{i}^{t}$	$\Delta C_{i}^{t}$	$\Delta G_{i}^{t}$	Status	
$\left(0,0.8\right)$	<0	<0	Weak negative decoupling	Negative decoupling
$\left(0,-\infty \right)$	>0	<0	Strong negative decoupling	
$\left(1.2,+\infty \right)$	>0	>0	Expansive negative decoupling	
$\left(1.2,+\infty \right)$	<0	<0	Recessive decoupling	
$\left(0,-\infty \right)$	<0	>0	Strong decoupling	Decoupling
$\left(0,0.8\right)$	>0	>0	Weak decoupling	
$\left(0.8,1.2\right)$	<0	<0	Recessive coupling	Coupling
$\left(0.8,1.2\right)$	>0	>0	Expansive coupling	

**Table 2:** Tapio elasticity coefficients and decoupling status of counties (by time period).

	Period	2001–2005	2006–2010	2011–2015	2016–2017	2001–2005	2006–2010	2011–2015	2016–2017
County									
Southern Jiangsu	Lishui	0.798	0.508	0.123	0.340	III	III	III	III
	Gaochun	0.792	0.517	0.066	0.791	III	III	III	III
	Jiangyin	1.492	0.570	−0.135	−0.268	I	III	IV	IV
	Yixing	1.162	0.620	0.001	−0.024	II	III	III	IV
	Liyang	1.054	0.573	−0.139	0.227	II	III	IV	III
	Jintan	0.820	0.457	0.153	0.183	II	III	III	III
	Changshu	1.435	0.539	0.006	−0.269	I	III	III	IV
	Zhangjiagang	1.178	0.487	0.100	−0.271	II	III	III	IV
	Kunshan	0.971	0.407	−0.126	−0.327	II	III	IV	IV
	Wujiang	1.716	0.576	0.003	0.011	I	III	III	III
	Taicang	1.761	0.679	−0.003	−0.257	I	III	IV	IV
	Danyang	1.268	0.615	−0.033	0.037	I	III	IV	III
	Yangzhong	1.213	0.815	0.036	0.661	I	II	III	III
	Jurong	1.065	0.564	0.046	0.367	II	III	III	III
Central Jiangsu	Hai’an	0.979	0.592	0.124	−0.319	II	III	III	IV
	Rudong	0.977	0.396	−0.042	−0.092	II	III	IV	IV
	Qidong	1.302	0.629	0.179	−0.060	I	III	III	IV
	Rugao	1.036	0.721	−0.050	−0.282	II	III	IV	IV
	Tongzhou	1.490	0.775	−0.099	−0.324	I	III	IV	IV
	Haimen	0.960	0.832	−0.069	−0.311	II	II	IV	IV
	Baoying	1.059	0.394	0.006	−0.281	II	III	III	IV
	Yizheng	1.066	0.602	0.022	−0.165	II	III	III	IV
	Gaoyou	1.090	0.481	0.020	−0.326	II	III	III	IV
	Jiangdu	1.054	0.502	−0.079	−0.133	II	III	IV	IV
	Xinghua	1.242	0.444	−0.099	−0.412	I	III	IV	IV
	Jingjiang	1.231	0.652	−0.061	0.273	I	III	IV	III
	Taixing	0.959	0.541	0.033	−0.005	II	III	III	IV
	Jiangyan	1.347	0.613	−0.071	0.604	I	III	IV	III
Northern Jiangsu	Fengxian	1.074	0.509	0.057	0.063	II	III	III	III
	Peixian	0.943	0.450	0.011	−0.020	II	III	III	IV
	Tongshan	0.986	0.407	0.022	−0.001	II	III	III	IV
	Suining	1.165	0.429	−0.009	−0.022	II	III	IV	IV
	Xinyi	1.086	0.588	−0.019	−0.045	II	III	IV	IV
	Pizhou	1.181	0.504	0.053	0.409	II	III	III	III
	Ganyu	2.440	0.419	−0.050	0.110	I	III	IV	III
	Donghai	1.592	0.542	−0.036	0.238	I	III	IV	III
	Guanyun	2.373	0.480	−0.040	0.021	I	III	IV	III
	Guannan	1.239	0.508	0.114	0.145	I	III	III	III
	Lianshui	1.211	0.529	−0.032	0.445	I	III	IV	III
	Hongze	1.117	0.409	0.018	−0.890	II	III	III	IV
	Xuyi	1.091	0.607	−0.171	−0.117	II	III	IV	IV
	Jinhu	1.142	0.447	−0.021	−0.236	II	III	IV	IV
	Xiangshui	1.031	0.506	0.233	−0.175	II	III	III	IV
	Binhai	1.168	0.394	−0.044	0.227	II	III	IV	III
	Funing	1.164	0.809	−0.164	−0.131	II	II	IV	IV
	Sheyang	1.145	0.515	−0.149	0.027	II	III	IV	III
	Jianhu	1.242	0.651	0.247	−0.134	I	III	III	IV
	Dongtai	1.014	0.543	−0.085	−0.918	II	III	IV	IV
	Dafeng	1.301	0.549	0.068	0.188	I	III	III	III
	Suyu	1.759	0.524	0.144	0.608	I	III	III	III
	Shuyang	1.129	0.487	−0.052	0.083	I	III	IV	III
	Siyang	1.431	0.378	−0.025	0.246	I	III	IV	III
	Sihong	1.102	0.495	−0.003	−0.206	II	III	IV	IV

I, II, III and IV represent expansive negative decoupling, expansive coupling, weak decoupling and strong decoupling, respectively.

**Table 3:** Full-sample panel cointegration regression results.

Variable	Mixed WLS Model	Fixed-Effects Model	Random-Effects Model
Constant	422.9690 ***	418.7417 ***	492.3611 ***
	(18.6029)	(17.9647)	(14.5626)
D	176.6310 ***	197.8404 ***	214.9132 ***
	(12.2936)	(16.2830)	(12.7201)
$r_{G}$	196.3138 ***	189.9356 ***	189.9142 ***
	(33.7408)	(29.4168)	(25.6826)
$r_{G}^{2}$	−4.9546 ***	−4.3557 ***	−3.3140 ***
	(−7.3921)	(−7.9765)	(−6.0156)
$D*r_{G}$	−33.5697 ***	−41.8320 ***	−36.1273 ***
	(−4.5887)	(−6.8112)	(−4.8796)
$D*r_{G}^{2}$	−1.5091 **	−1.2341 **	−1.9856 **
	(−2.0105)	(−2.1831)	(−2.3130)
S	−8.6781 ***	−8.9019 ***	−10.9278 ***
	(−12.8355)	(−12.2186)	(−10.7589)
F	0.2229	1.4847 ***	0.6394 ***
	(1.6382)	(6.4250)	(3.2516)
E	−6.7956 ***	−4.8176 ***	−5.0504 ***
	(−17.9484)	(−11.0578)	(−7.8850)
Er	4.5676 ***	1.2081	0.5690
	(3.8252)	(1.0691)	0.3667
R2	0.9377	0.9645	0.9041
Adjusted R2	0.9371	0.9621	0.9032
F statistic	1575.634 ***	396.872 ***	986.743 ***
Curve type	Inverted U	Inverted U	Inverted U
Turning point $r_{G}$ (10,000 yuan)	12.5895	13.2478	14.5095
F test	22.3446 ***		
Hausman test	24.7455 ***		

*** and ** indicate passing the significance test of 1% and 5%; *t*-values are reported in parentheses; the turning point refers to the EKC of 2009–2017.

**Table 4:** Two sub-sample panel cointegration regression results.

Variable	Low-Income Group Fixed-Effects Model	High-Income Group Random-Effects Model
Constant	295.1312 ***	571.9830 ***
	(10.9210)	(7.6791)
D	231.3894 ***	105.7127 ***
	(11.7652)	(5.2503)
$r_{G}$	346.0248 ***	176.3787 ***
	(15.2585)	(21.9752)
$r_{G}^{2}$	−46.8190 ***	−3.0664 ***
	(−6.2455)	(−4.8904)
$D*r_{G}$	−159.6846 ***	
	(−7.1853)	
$D*r_{G}^{2}$	34.3327 ***	−3.0840 ***
	(4.8990)	(−9.5510)
S	−7.9037 ***	−12.8276 ***
	(−9.7864)	(−5.5279)
F	0.0162	1.0441 ***
	(0.0286)	(4.2998)
E	−1.4830 ***	−6.5716 ***
	(−3.0790)	(−10.4134)
Er	2.2764 *	−3.7016
	(1.8705)	(−1.4017)
R2	0.9511	0.9739
Adjusted R2	0.9476	0.9715
F statistic	269.8116 ***	408.9470 ***
Curve type	Inverted U	Inverted U
Turning point $r_{G}$ (10,000 yuan)	7.4618	14.3387
F test	25.9423 ***	29.0179 ***
Hausman test	51.9276 ***	15.9530 **

***, ** and * indicate passing the significance test of 1%, 5% and 10%; *t*-values are reported in parentheses; the turning point refers to the EKC of 2009–2017.

## Data Availability

The data presented in this study are available on request from Jiangsu Statistical Yearbook, its prefecture-level city Statistical Yearbook from 2001 to 2018, and the CEADs database (http://www.ceads.net.cn) (accessed on 18 February 2020).

## Appendix A

**Table A1:** Regional division of the counties.

Regional Division	Counties
Southern Jiangsu	Lishui, Gaochun, Jiangyin, Yixing, Liyang, Jintan, Changshu, Zhangjiagang, Kunshan, Wujiang, Taicang, Danyang, Yangzhong and Jurong
Central Jiangsu	Hai’an, Rudong, Qidong, Rugao, Tongzhou, Haimen, Baoying, Yizheng, Gaoyou, Jiangdu, Xinghua, Jingjiang, Taixing and Jiangyan
Northern Jiangsu	Fengxian, Peixian, Tongshan, Suining, Xinyi, Pizhou, Ganyu, Donghai, Guanyun, Guannan, Lianshui, Hongze, Xuyi, Jinhu, Xiangshui, Binhai, Funing, Sheyang, Jianhu, Dongtai, Dafeng, Suyu, Shuyang, Siyang and Sihong

**Table A2:** Descriptive statistical analysis.

	Region	Jiangsu	Southern Jiangsu	Central Jiangsu	Northern Jiangsu
Variable					
$r_{C}$ (ton/100 person)	Mean	641.408	939.251	625.605	483.466
	CV	0.574	0.444	0.503	0.508
$r_{G}$ (10,000 yuan/person)	Mean	3.427	6.430	3.000	1.984
	CV	0.905	0.604	0.702	0.760
$r_{G}^{2}$ (10,000 yuan/person)^2^	Mean	21.359	56.359	13.420	6.204
	CV	1.852	1.090	1.302	1.492
S (%)	Mean	36.116	38.067	37.188	34.424
	CV	0.151	0.118	0.134	0.166
F (%)	Mean	22.106	56.241	17.701	5.419
	CV	1.902	1.241	0.602	0.729
E (yuan/kWh)	Mean	9.164	9.812	9.526	8.598
	CV	0.703	0.931	0.737	0.423
Er (%)	Mean	0.270	0.328	0.228	0.261
	CV	0.115	0.107	0.110	0.123

CV, coefficient of variation.

**Table A3:** Tapio elasticity coefficients of Jiangsu and three regions (2001–2017).

Year	Jiangsu	Southern Jiangsu	Central Jiangsu	Northern Jiangsu
2001	0.024	0.081	−0.029	0.003
2002	0.935	1.195	0.922	0.777
2003	1.476	1.551	1.430	1.802
2004	0.755	0.746	0.759	0.874
2005	1.249	1.208	1.338	1.326
2006	0.850	0.864	0.915	0.866
2007	0.605	0.569	0.756	0.576
2008	0.590	0.639	0.671	0.468
2009	0.455	0.451	0.450	0.455
2010	0.733	0.751	0.690	0.733
2011	1.132	1.026	1.202	1.157
2012	0.181	0.100	0.233	0.210
2013	−0.039	−0.080	−0.010	−0.023
2014	0.206	0.288	0.066	0.226
2015	−0.479	−0.438	−0.459	−0.482
2016	0.317	0.348	0.244	0.334
2017	−0.060	−0.072	−0.147	0.025

**Table A4:** Tapio elasticity coefficients of Jiangsu and three regions (by time period).

Period	Jiangsu	Southern Jiangsu	Central Jiangsu	Northern Jiangsu
2001–2005	1.126	1.226	1.136	1.212
2006–2010	0.546	0.550	0.597	0.507
2011–2015	−0.015	−0.016	−0.026	−0.004
2016–2017	−0.060	−0.072	−0.147	0.025

**Table A5:** Unit root tests of level and first-order difference series.

Variable		LLC	Breitung	IPS	ADF Fisher	PP Fisher
$r_{C}$	Level	11.2377	6.7416	10.8931	15.0334	15.0631
	First-order Difference	−13.4367 ***	−3.5786 ***	−4.1365 ***	155.286 ***	265.496 ***
$r_{G}$	Level	7.9309	16.5135	−0.1803	90.7183	82.8784
	First-order Difference	−4.8106 ***	−11.2595 ***	−2.2889 **	159.619 ***	190.095 ***
$r_{G}^{2}$	Level	10.4986	15.3340	12.0527	46.6505	2.0181
	First-order Difference	−13.6045	−0.4918	−7.9376 ***	253.008 ***	184.044 ***
S	Level	1.7683	3.3884	0.7803	94.8114	50.5750
	First-order Difference	−8.7172 ***	−3.1150 ***	−3.1725 ***	157.545 ***	263.396 ***
F	Level	0.6618	3.3884	0.7803	94.8114	50.5750
	First-order Difference	−20.7784 ***	−3.1150 ***	−3.1725 ***	157.545 ***	263.396 ***
E	Level	10.3054		4.7888	83.1245	109.432
	First-order Difference	−2.2914 **		−9.1425 ***	332.920 ***	474.983 ***
Er	Level	44.2992	8.0069	3.5978	74.4350	75.3880
	First-order Difference	−20.8454 ***	−7.3702 ***	−9.2766 ***	152.301 ***	557.747 ***

In combination with the trend of the series, E selects the test model containing the constant terms (therefore, the Breitung test cannot be carried out), and the other 6 series are suitable for the test model containing constant and trend terms. *** and ** indicate passing the significance test of 1% and 5%.

**Table A6:** Panel cointegration tests.

Test Method	Statistic	Statistics Value	*p*-Value
Pedroni Test	Panel v Statistic	0.8587 **	0.0445
	Panel rho Statistic	4.7534	1.0000
	Panel PP Statistic	−0.6267 ***	0.0003
	Panel ADF Statistic	−1.7245 ***	0.0001
	Group rho Statistic	6.7631	1.0000
	Group PP Statistic	−4.1039 ***	0.0000
	Group ADF Statistic	−3.4725 ***	0.0003
Kao Test	ADF	−6.2063 ***	0.0000
Johansen Fisher Test	Fisher Statistic	3323 ***	0.0000

*** and ** indicate passing the significance test of 1% and 5%, respectively; and Johansen Fisher test only lists the case without cointegration.
